# When to Use Medicine in Pneumonia

**Published:** 1907-02

**Authors:** 


### WHEN TO USE MEDICINE IN PNEUMONIA

---

Dudley Morgan contributes a paper to the Medical Record under this title, which, he points out, suggests, as he means it shall, that there are some cases of pneumonia requiring nothing but intelligent and systematic guidance and nursing, and other needing little medicine, but needing it promptly and energetically when called for.

It is a matter of surprise sometimes how well some individuals bear pneumonia. We have all had patients who have successfully weathered the storm in two or three attacks. Before beginnig treatment, as well as before giving a prognosis, it is well to try and balance up the good and bad conditions which confront us. What is the condition of the heart, the kidneys, the liver? Is the patient tuberculous or does pregnancy complicate the case, etc.? It takes very little in pneumonia to cause us to be heavily handicapped. When the disease occurs in either extreme of life; the infant's life may be cut off in a convulsion, the octogenarian's may go out like a flash. A profund pneumonic toxemia will call forth all our aid to combat and woe to the patient if his life has been spent more at table d'hote and with the wine merchant than at the frugal and simple table. There is no more unsatisfactory class of cases to attend than those who have worshipped Bacchus as one of their gods and fall a victim to pneumonia. Pneumonia is often the Good Samaritan to the enebriate and the benefactor to his friends, as it quickly and with short pain hastens him into the coma of rest and cuts short a career which could bring but sorrow and suffering to his family and friends.

Given digatalis, strychnine, and ice with the various uses, and you need little else even in your most trying cases. There is no question that many cases are lost from too much meddling in many ways. How often do we start strychnine on our first visit for no other reason than perhaps it may be needed the next day, or that we think we must give something. Quiet and rest, good ventilation, unloading of the bowels, a variety of liquid nourishing food, the ice bag to the chest in the region of the pain and congestion and one also over the precordia, if need be, is a good way to start with nearly all cases of pneumonia. 'Tis granted that there are some cases of the sthenic type, with bounding and laboring heart, high fever, pain and restlessness, and great dyspnea, in which the withdrawal of blood has been the only thing which would save life. Other most trying and often unsatisfactory cases to treat are those of pneumonia, as has been referred to, in the steady or hard drinker. Here the heart may begin to lag almost before apparent congestion and consolidation; the pulse become soft and compressible, the face ashy, the patient is restless, soon delirious, the temperature may not be high, but there is an impending toxemia which is and will continue to show itself in weakening and overpowering the action of the heart and the vasomotor centers. Such cases as these need digitalis and strychnine early to support the blood-vessels, bromides to quiet the restlessness, and salt solutions by hyperdermoclysis or enemata to raise the blood pressure and dilute the toxins. There is no more satisfactory way of keeping up proper elimination and sustaining blood pressure than the use of frequent saline solutions. There are many cases of pneumonia complicated with parenchymatous nephritis, which one feels quite confident were saved through his treatment.

The writer has a great repugnance to the use of opium in pneumonia, and feels that most of the cases he has lost may have been from the injudicious use of opium. There is little more needed for the cough and pain than the ice bag, but should there be, and the patient is, besides, restless, it is seldom necessary to give more than the one-twenty-fourth of a grain of morphine with fifteen grains of bromide once or twice in twenty-four hours.

In an extensive hospital ward practice many of the remedies which are suggested at the present day have been used only to be soon abandoned. There is no question that creosote may have some little palliative effect upon the cough, through the nerve filaments of the mucous menbrane, that it may render the secretions more or less antiseptic and produce other slight changes, but as for any curative or abortive effect it is wasting time to consider it. To the use of nitroglycerin in certain stages of the disease, one cannot be opposed. There is a time when the right heart is laboring under the load before it in the lung, and there is an impending venous stasis, when the use of nitroglycerin or light bleeding and the giving of saline infusions have dilated the vasomotors and so relieved the heart and at the same time conserved the strength of the patient. Oxygen if needed should be used more to quiet the patient in his dyspnea and so enable him to get short naps and avoid the use of morphine. Veratrum viride has its advocates, but the good it does, if any, is not nearly so apparent in pneumonia as in typhoid or some of the fevers of the puerperium, where its beneficial use has long been established.

It is but natural, when we remember that the mortality is as great now as fifty years ago, that new methods of treatment should be proposed and heralded; the quinine-iron treatment has its backers, Page's system of fasting has had a run. The cold-tar treatment of a few years ago is hardly ever heard of; it had as its only recommendation as Dr. Simon Baruch said, that the patient died with a fairly normal temperature. We hoped for great things from the antipneumococcic serum, but results are as yet not convincing.

It is thus that we hold to digitalis and strychnine, and are chary at adopting other drugs and their combination, believing, as Niemeyer and Jaccoud have often spoken and written, that digitalis should be used to strengthen and nourish the heart in pneumonia and reduce a rapid pulse. The words of Thomas of London in his practice in 1813 on digitalis can appropriately be repeated in our day: "Use your digitalis in pneumonia to strength and diminish the action of the heart."—*Medical Standard, Jan.*, '07.

